# Impact of sleep debt, social jetlag, and insomnia symptoms on presenteeism and psychological distress of workers in Japan: a cross-sectional study

**DOI:** 10.1186/s13030-022-00242-5

**Published:** 2022-06-03

**Authors:** Yuta Takano, Rui Ibata, Norihito Nakano, Yuji Sakano

**Affiliations:** 1 Department of Psychology, Fukuyama University, 985-1 Sanzo, Higashimura-Cho, Fukuyama, Hiroshima 729-0292 Japan; 2 Graduate School of Psychological Science, Health Sciences University of Hokkaido, Hokkaido, Japan; 3 Goryokai Medical Corporation, Hokkaido, Japan; 4 School of Psychological Science, Health Sciences University of Hokkaido, Hokkaido, Japan; 5 CBT & EAP Center, Goryokai Medical Corporation, Hokkaido, Japan

**Keywords:** Sleep debt, Social jetlag, Insomnia symptoms, Presenteeism, Cross-sectional survey

## Abstract

**Background:**

Presenteeism is an indicator of productivity loss and the risk of absence from work due to mental health problems. The purpose of this study was to determine the impact of sleep debt, social jetlag, and insomnia symptoms on presenteeism and psychological distress.

**Methods:**

The participants were 351 Japanese workers (271 males, 79 females, and one of other gender, with a mean age of 49 ± 9.49 years). The eligibility criteria for this study were full-time employment, working eight hours per day, five days per week, and no night shifts. The participants answered questionnaires measuring sleep debt, social jetlag, insomnia symptoms, presenteeism, and psychological distress.

**Results:**

Insomnia symptoms had the greatest impact on presenteeism and psychological distress when compared with sleep debt and social jetlag (adjusted odds ratio (OR) = 5.61, 95% confidence interval (CI) = 2.88–10.91; adjusted OR = 7.29, 95%CI = 3.06–17.35). Sleep debt had a greater impact on presenteeism and psychological distress than did social jetlag (adjusted OR = 1.61, 95%CI = 1.14–2.27; adjusted OR = 1.68, 95%CI = 1.11–2.54), which had no impact on these variables (adjusted OR = 1.04, 95%CI = 0.91–1.20; adjusted OR = 0.96, 95%CI = 0.76–1.22).

**Conclusions:**

The findings of this study indicated that insomnia symptoms had a more significant impact on presenteeism and psychological distress than social jetlag and sleep debt. Although sleep debt might have an independent impact on presenteeism and psychological distress, social jetlag did not.

## Background

The average short sleep duration of Japan’s working population 30–60 years [1] contributes significantly to productivity and economic loss caused by lack of sleep [2]. Presenteeism is an indicator of productivity loss characterized by health problems even when workers are present at work [3]. Presenteeism is also a predictor of absence due to mental health problems [4].

Sleep debt is a concept that indicates the total amount of sleep lost by an individual [5]. Sleep debt worsened presenteeism on linear distribution and indicated that absolute sleep duration does not reflect the amount of sleep an individual required [6]. Sufficient sleep duration refers to the amount of sleep an individual requires, and obtaining the necessary amount of sleep duration is more essential than the absolute duration [6]. Therefore, in the relationship between sleep duration and presenteeism, it is important to focus on sleep debt rather than absolute sleep duration.

When there are social constraints, such as regular work hours, the time of waking up is determined by social time, such as the time of arrival at work, resulting in a discrepancy with the individual's circadian rhythm. This discrepancy is called social jetlag, which can be quantified as the absolute difference between mid-sleep on workdays and weekends [7]. Social jetlag may be calculated only by the difference between weekday and weekend midsleep points and may have a negative value if the weekend midsleep point is earlier than the weekday midsleep point. However, the percentage of social jetlag having a negative value is very low [8, 9]. In addition, absolute differences are most often used when considering the impact of social jetlag [10] See supplemental information]. For example, absolute differences are used in the association between social jetlag and depressive symptoms [11, 12]. Absolute differences are also used in relation to chronotype and social jetlag [8]. Therefore, this study also used the absolute difference for the evaluation of social jetlag. Social jetlag has been associated with depressive symptoms that may be independent of sleep debt [6]. Hence, social jetlag has been associated with presenteeism, in which productivity is impaired due to health problems; however, this association has not been fully clarified [6, 13]. A previous study reported that social jetlag’s effect on presenteeism was masked by sleep debt, but the study excluded individuals with negative social jetlag [6]. Therefore, it is necessary to examine whether social jetlag is a factor that explains presenteeism, independent of sleep debt.

The relationship between insomnia symptoms, presenteeism, sleep debt, and social jetlag has also been examined. Insomnia symptoms are considered an aggravating factor for presenteeism [14]. In a study of Japanese workers, those with insomnia symptoms also had higher rates of presenteeism than those without insomnia symptoms [15, 16].

Sleep problems impact presenteeism, but it is unclear which factors among sleep debt, social jetlag, or insomnia symptoms have the most significant impact. In addition, sleep debt, social jetlag, and insomnia symptoms are also associated with mental health problems [6, 17]. Mental health problems were shown to worsen presenteeism [18] and presenteeism predicted mental health problems one year later [19], indicating that mental health problems and presenteeism are interrelated. Therefore, a simultaneous examination of presenteeism and mental health problems would provide noteworthy information on the mental health of workers and their daytime function. The purpose of this study was to determine the impact of sleep debt, social jetlag, and insomnia symptoms on presenteeism and psychological distress.

## Methods and materials

### Study design, participants, and procedure

**Fig. 1:**
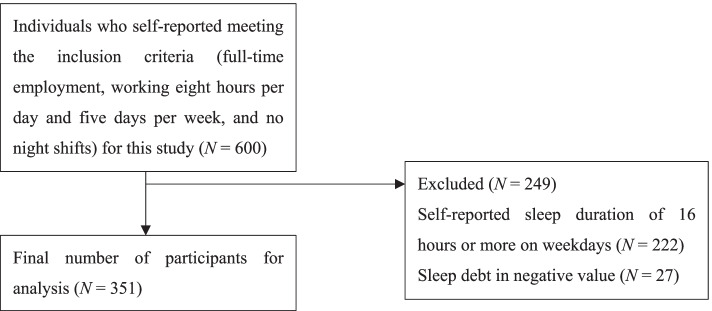
Flowchart of the participant selection for this study

This study used a cross-sectional design by conducting a web-based survey through an internet research company (Cross Marketing Inc.). Cross Marketing's active panel for the most recent year was 2.95 million. Individuals between the ages of 20–69 who had registered with the research company were randomly selected and requested to complete the survey. The following screening criteria were used for inclusion: full-time employment, working eight hours per day and five days per week, and no night shifts. A total of 600 workers were included in the study. Participants with a negative sleep debt index were excluded following previous studies [6]. A negative value for the sleep debt index is not the definition of sleep debt [5]. Participants with more than 16 h of sleep duration on weekdays were excluded. The final number of participants was 351 (271 males, 79 females, and 1 of other gender). The mean age was 49 ± 9.49 years. The selection process for the participants is shown in Fig. 1. The survey was conducted from June 18–June 21, 2021.

All the procedures performed in this study were in accordance with the ethical standards of the institutional and national research committee and with the 1964 Helsinki Declaration. This study was approved by the Ethical Board of the School of Psychological Sciences, Health Sciences University of Hokkaido, Japan (No. 21004), and the Research Ethics Committee of Fukuyama University, Japan (No. 2021-H-8).

### Measures

The Sleep Debt Index (SDI) is a self-report scale to assess sleep debt. Actual total sleep times were calculated by adding up weekday and weekend total sleep times and dividing them by seven days. The SDI was then calculated by subtracting the actual total sleep time from the individual's self-reported ideal total sleep time [6].

The Japanese version of the Munich ChronoType Questionnaire [20] is a self-report scale to assess social jetlag. Social jetlag was calculated as the absolute difference between mid-sleep on workdays and weekends [7].

The Japanese version of the Insomnia Severity Index (ISI–J) is a self-report scale to assess insomnia symptoms. The ISI–J is considered reliable and valid, with a cutoff value of ten points [21]; an ISI–J score of ten or more points indicates the presence of insomnia symptoms.

The World Health Organization Health and Work Performance Questionnaire, Japanese version (WHO–HPQ) is a self-report scale to assess presenteeism and absenteeism [22]. The WHO-HPQ can evaluate presenteeism as absolute and relative presenteeism. In this study, only absolute presenteeism was used. In the development study of the WHO-HPQ, it was shown that the self-evaluation of absolute presenteeism assessed by the WHO-HPQ is consistent with the supervisor's evaluation of the individual's work performance [23]. The items for evaluating absolute presenteeism are the performance of work over the past four weeks, using an 11-point Likert scale from 0 (worst performance) to 10 (best performance). The absolute presenteeism score was then calculated by multiplying the crude score by 10. The cutoff value of the WHO–HPQ is established at 40 points, and absence due to mental health concerns increases when the score is 40 or less [19]. Therefore, in this study, a WHO–HPQ score of 40 or less indicated presenteeism.

The Japanese version of the K6 is a self-report scale to assess psychological distress. The K6 is a valid measure [24], and scores of 13 or more points indicate the presence of psychological distress [25].

### Statistical analysis

R 3.6.1 was used for statistical analysis. Shapiro–Wilk test was performed to confirm the normality of the data. Spearman's rank correlation coefficient was used for correlations between variables. The *glm* function was used for the multiple logistic regression analysis. The R package’s *epiDisplay* [26], *ResourceSelection* [27], and *car* [28] were used. The variables used in multiple logistic regression analyses were coded. Presenteeism (WHO–HPQ scores ≤ 40, *N* = 49) was coded as 1, and no presenteeism (WHO–HPQ scores > 40, *N* = 302) was coded as 0. Psychological distress (K6 scores ≥ 13, *N* = 30) was coded as 1, and no psychological distress (K6 scores < 13, *N* = 321) was coded as 0. Insomnia symptoms (ISI–J scores ≥ 10, *N* = 103) were coded as 1, and no insomnia symptoms (ISI–J scores < 10, *N* = 248) were coded as 0. Sleep debt and social jetlag were used as continuous variables. To address the problem of overfitting, the number of independent variables that could be entered into a multiple logistic regression analysis was calculated. Forty-nine participants were classified as having presenteeism, and 30 participants were classified as having psychological distress. To avoid overfitting, it is recommended that the number of events in the dependent variable divided by the independent variable be greater than 10 [29]. Therefore, only three independent variables were used: sleep debt, social jetlag, and insomnia symptoms.

In multiple logistic regression analysis, the odds ratio (OR) and 95% confidence interval (CI) of presenteeism and psychological distress were estimated with sleep debt, social jetlag, and insomnia symptoms as independent variables. In both analyses, with presenteeism as the dependent variable and psychological distress as the dependent variable, independent variables were entered in a simultaneous imputation method with sleep debt and social jetlag in Model 1 and sleep debt, social jetlag, and insomnia symptoms in Model 2. A previous study indicated that social jetlag did not affect presenteeism based on the results of a multiple regression analysis of the two variables of sleep debt and social jetlag [6].

Multicollinearity was checked using the variance inflation factor (VIF). A VIF of ten or more was considered to indicate a multicollinearity problem. The Hosmer–Lemeshow goodness-of-fit test was used to evaluate the fit of the models.

## Results

The results of the Shapiro–Wilk test did not confirm normality for all variables. Descriptive statistics are presented in Table 1. The results of the correlation analysis of sleep debt, social jetlag, insomnia symptoms, presenteeism, and psychological distress are shown in Table 2.

**Table 1:** Participants’ demographic data and descriptive statistics for each variable

	*M*	*SD*	*Median*	Min	Max
Age	49	9.49	50	22	68
BMI	22.95	3.65	22.4	13.89	40.12
Sleep debt (h)	1.01	0.84	1	0	5.14
Social jetlag (h)	1.18	1.87	0.75	0	19.00
Insomnia symptoms	7.75	5.38	7	0	28
Presenteeism	58.86	16.52	60	0	100
Psychological distress	4.55	5.60	2	0	24

*Note*. *M* Mean, *SD* Standard deviation, *BMI* Body Mass Index

**Table 2:** Results of Spearman's rank correlation analysis of sleep debt, social jetlag, insomnia symptoms, presenteeism, and psychological distress

	Sleep debt	Social jetlag	Insomnia symptoms	Presenteeism	Psychological distress
Sleep debt	–				
Social jetlag	0.19^*^	–			
Insomnia symptoms	0.31^*^	0.08	–		
Presenteeism	-0.21^*^	-0.07	-0.43^*^	–	
Psychological distress	0.22^*^	0.06	0.55^*^	-0.38^*^	–

*Note*. ^*^ *p* < .05

The occupational categories of the participants were as follows: office worker, public servant or teacher or non-profit organization worker, self-employed, small office/home office (SOHO), professional, and other occupations, accounting for 82.3%, 9.4%, 4.3%, 0.9%, 2.8%, and 0.3%, respectively.

OR and 95%CI estimated with presenteeism as the dependent variable are presented in Table 3. In Model 1, in which sleep debt and social jetlag were entered as independent variables, sleep debt significantly impacted presenteeism (adjusted OR = 1.61, 95%CI = 1.14–2.27), while social jetlag was not (adjusted OR = 1.04, 95%CI = 0.91–1.20). The VIF for sleep debt and social jetlag were 1.02 and 1.02, respectively, and there were no multicollinearity problems. The Hosmer–Lemeshow goodness-of-fit test confirmed that the model fit well (*p* = 0.21). In Model 2, in which sleep debt, social jetlag, and insomnia symptoms were entered as independent variables, sleep debt and social jetlag did not significantly impact presenteeism (adjusted OR = 1.30, 95%CI = 0.90–1.89; adjusted OR = 1.08, 95%CI = 0.93–1.25); however, insomnia symptoms did (adjusted OR = 5.61, 95%CI = 2.88–10.91). The VIF for sleep debt, social jetlag, and insomnia symptoms were 1.05, 1.02, and 1.05, respectively, and there were no multicollinearity problems. The Hosmer–Lemeshow goodness-of-fit test confirmed that the model fit well (*p* = 0.55).

**Table 3:** Multiple logistic regression analysis results of presenteeism and sleep debt, social jetlag, and insomnia symptoms

	Model 1				Model 2			
Variable	Estimate	*SE*	Crude OR (95% CI)	Adjusted OR (95% CI)	Estimate	*SE*	Crude OR (95% CI)	Adjusted OR (95% CI)
Sleep debt (one hour increase)	0.48^*^	0.18	1.63 (1.16 – 2.29)	1.61 (1.14 – 2.27)	0.27	0.19	1.63 (1.16 – 2.29)	1.30 (0.90 – 1.89)
Social jetlag (one hour increase)	0.04	0.07	1.07 (0.94 – 1.22)	1.04 (0.91 – 1.20)	0.07	0.07	1.07 (0.94 – 1.22)	1.08 (0.93 – 1.25)
Insomnia symptoms					1.72^*^	0.34	6.12 (3.12 –11.68)	5.61 (2.88 –10.91)

*Note*. *SE* Standard error, *OR* Odds ratio, *CI* Confidence interval, ^*^ *p* < 0.01

In both Model 1 and Model 2, the independent variables were entered in a simultaneous imputation method

OR and 95%CI estimated with psychological distress as the dependent variable are shown in Table 4. In Model 1, in which sleep debt and social jetlag were entered as independent variables, sleep debt significantly impacted psychological distress (adjusted OR = 1.68, 95%CI = 1.11–2.54), but social jetlag was not (adjusted OR = 0.96, 95%CI = 0.76–1.22). The VIF of sleep debt and social jetlag were 1.04 and 1.04, respectively, and there was no multicollinearity problem. The Hosmer–Lemeshow goodness-of-fit test confirmed that the model fit well (*p* = 0.65). In Model 2, in which sleep debt, social jetlag, and insomnia symptoms were entered as independent variables, sleep debt and social jetlag did not significantly impact psychological distress (adjusted OR = 1.31, 95%CI = 0.84–2.04; adjusted OR = 0.99, 95%CI = 0.77–1.27), however, insomnia symptoms did (adjusted OR = 7.29, 95%CI = 3.06–17.35). The VIF for sleep debt, social jetlag, and insomnia symptoms were 1.07, 1.03, and 1.04, respectively, with no multicollinearity problems. The Hosmer–Lemeshow goodness-of-fit test confirmed that the model fit well (*p* = 0.82).

**Table 4:** Multiple logistic regression analysis of psychological distress and sleep debt, social jetlag, and insomnia symptoms

	Model 1				Model 2			
Variable	Estimate	*SE*	Crude OR (95% CI)	Adjusted OR (95% CI)	Estimate	*SE*	Crude OR (95% CI)	Adjusted OR (95% CI)
Sleep debt (one hour increase)	0.52^*^	0.21	1.66 (1.10 – 2.48)	1.68 (1.11 – 2.54)	0.27	0.23	1.66 (1.10 – 2.48)	1.31 (0.84 – 2.04)
Social jetlag (one hour increase)	-0.04	0.12	1.00 (0.82 – 1.22)	0.96 (0.76 – 1.22)	-0.00	0.13	1.00 (0.82 – 1.22)	0.99 (0.77 – 1.27)
Insomnia symptoms					1.99^*^	0.44	8.15 (3.49 – 19.02)	7.29 (3.06 – 17.35)

*Note*. *SE* Standard error, *OR* Odds ratio, *CI* Confidence interval, ^*^ *p* < 0.01

In both Model 1 and Model 2, the independent variables were entered in a simultaneous imputation method

## Discussion

The purpose of this study was to clarify the impact of sleep debt, social jetlag, and insomnia symptoms on presenteeism and psychological distress. The findings indicated that sleep debt had a more significant impact on presenteeism and psychological distress than social jetlag. Among sleep debt, social jetlag, and insomnia symptoms, insomnia symptoms had a more significant impact than the other two variables on presenteeism and psychological distress. Thus, insomnia symptoms had the more significant impact on both presenteeism and psychological distress, and sleep debt had a more substantial impact than social jetlag. The impact on presenteeism and psychological distress was most significant for insomnia symptoms, followed by sleep debt. Therefore, it is essential to address insomnia symptoms. On the other hand, for those with sleep debt and no insomnia symptoms, the sleep debt needs to be addressed. However, no treatment program has been designed to improve presenteeism by addressing sleep debt [30]. This study is meaningful because it highlights the importance of addressing insomnia symptoms and sleep debt and the need to develop a program for sleep debt.

The impact of sleep debt on presenteeism was more significant than that of social jetlag, a finding that is consistent with previous research [6]. In the National Health and Nutrition Survey conducted in 2019, 37.5% of men and 40.6% of women slept less than six hours [31]. Compared to those who sleep seven to eight hours, those who sleep six hours or less have a higher rate of presenteeism [32]. Moreover, Japan faced the world's worst economic loss due to sleep debt [2]. Therefore, it is likely that sleep debt has affected presenteeism. In an experiment using a psychomotor vigilance task after two weeks of sleep restriction, the response time to the task gradually increased in the group that was restricted to four to six hours of sleep [33]. Although performance on cognitive functioning tasks decreases with sleep restriction, individuals do not perceive subjective sleepiness and follow a subjective sense of adaptability, even in the presence of chronic sleep restriction [33]. Therefore, people with sleep debt underestimate presenteeism, which is considered a daytime dysfunction. In modern society, it has been observed that people sleep longer on weekends than on weekdays [8], and many workers have a sleep debt. Social jetlag affects productivity in the group with short sleep duration but does not affect productivity in the group with long sleep duration, even when the social jetlag is large [34]. The results indicate that longer sleep duration may be a protective factor against productivity loss due to social jetlag [34]. In other words, although sufficient sleep duration on workdays is vital to reduce the impact on presenteeism, when achieving this is challenging, it is essential to sleep longer on weekends, even if this causes social jetlag. The results of the present study indicate that focusing on improving sleep debt or ensuring that individuals get the amount of sleep they need is important for improving presenteeism.

Social jetlag is a discrepancy between social time and the individual's circadian rhythm and may be excluded as it cannot be accurately assessed in people who use alarm clocks on weekends [8]. On the other hand, this study did not exclude individuals who use alarm clocks on weekends. This is becsuse the study also assesses sleep debt. Social jetlag is calculated as the discrepancy between the midpoint of sleep on weekdays and weekends. In general, workers sleep longer on weekends than on weekdays [8]. This suggests that sleep debt is present. Excluding those who used alarm clocks on weekends might exclude those with sleep debt. Japan has the world's worst economic loss due to sleep debt [2], and both objective and subjective sleep duration is short [1, 31]. For this reason, the evaluation of sleep debt was prioritized over the evaluation of social jetlag. The social jetlag in the previous study was 0.91 h on average [8], and the social jetlag in the present study was similar at 1.18 h on average. Therefore, while there may be some concern in terms of accurately assessing social jetlag, it is unlikely to affect the findings.

Insomnia symptoms had a more significant impact on presenteeism than sleep debt and social jetlag. Insomnia symptoms can be an aggravating factor for presenteeism, and the OR for presenteeism was reported at 5.49 in those with insomnia symptoms compared to those without such symptoms [14], which is consistent with the results of the present study. Therefore, this study confirms that insomnia symptoms affect presenteeism. Insomnia symptoms might have had the most significant impact on presenteeism due to persistence; research has shown persistence of insomnia symptoms at one, three, and five years of follow-up [35]. When insomnia was present at baseline, its persistence was observed in 70.7% of participants at one year, 49.7% at three years, and 37.5% at five years. In another study, its persistence was found in 86.0% at one year, 72.4% at three years, and 59.1% at five years in the presence of sleep-related psychological distress or daytime dysfunction problems [35]. Some of those with insomnia symptoms additionally present daytime dysfunction. Therefore, those with insomnia symptoms may be more likely to experience presenteeism, which is considered a daytime dysfunction. Given the above, insomnia symptoms may have more significantly impacted presenteeism than sleep debt.

Insomnia symptoms had the most significant impact on psychological distress, while sleep debt had a more substantial impact on psychological distress than social jetlag. Because insomnia symptoms [17] and short sleep duration are both risk factors for depression [36], the results of the present study are valid. Although social jetlag has been reported to increase depressive symptoms [6], the results of the present study differed from those of previous studies. A previous study used the Center for Epidemiologic Studies Depression (CES-D) scale, but the mean score was very low, which may be insufficient to identify the presence of depressive symptoms [6]. However, this study did not show that social jetlag affects psychological distress equivalent to insomnia symptoms and sleep debt. This does not imply that social jetlag need not be addressed but that it is less critical than insomnia symptoms and sleep debt.

There are several limitations to this study. First, although the study found that sleep debt and insomnia affected presenteeism and psychological distress independently, it failed to take into account the possibility that some people would have both insomnia and sleep debt at the same time. One of the characteristics of those with insomnia is that they rate their subjective sleep duration as shorter than their actual sleep duration when their objective sleep duration is six hours or more. However, when their objective sleep duration is less than six hours, their subjective sleep duration is never shorter than their actual sleep duration [37]. In other words, people with an objective sleep duration of more than six hours and insomnia symptoms may overestimate their sleep debt. Future studies need to use objective and subjective sleep measures and to include those who have both insomnia and sleep debt. Second, this study was conducted using an Internet survey. The participants in this study were those sufficiently interested in sleep and healthy to complete the Internet survey. Because sleep debt and insomnia symptoms are concerns in Japan and worldwide, replication of the results of this study in other studies would enhance the reliability of the results. Third, the design of this study prioritized the evaluation of sleep debt over social jetlag. Hence, those who used alarm clocks on weekends were not excluded from the study. However, the evaluation of social jetlag may have been compromised because those who used an alarm clock on weekends were not excluded. It should be cautioned that, although this study found that sleep debt had a greater impact on presenteeism and psychological distress than social jetlag, this result may be because of the study design.

## Conclusions

This study demonstrated that insomnia symptoms had a more significant impact on presenteeism and psychological distress than did sleep debt and social jetlag, indicating for the first time that insomnia symptoms are the most critical sleep problem to be addressed. In addition, limited results indicated that sleep debt had a greater impact on presenteeism and psychological distress than did social jetlag. To this end, assessment of insomnia symptoms and sleep debt plays a vital role in addressing productivity loss and mental health problems among workers.

## Data Availability

The data cannot be shared because permission to do so was not obtained from the participants at the time of the survey.

## Abbreviations

SDI: Sleep Debt Index; ISI–J, The Japanese version of the Insomnia Severity Index
WHO–HPQ: World Health Organization Health and Work Performance Questionnaire Japanese version
K6: Japanese version of the K6
OR: Odds ratio
CI: Confidence interval
VIF: Variance inflation factor
SOHO: Small office/home office
CES-D: Center for Epidemiologic Studies Depression

## References

[1] Li L, Nakamura T, Hayano J, Yamamoto Y. Age and gender differences in objective sleep properties using large-scale body acceleration data in a Japanese population [Sci. rep.:9970] [Sci. rep.:9970]. Sci Rep. 2021;11:9970. 10.1038/s41598-021-89341-x 10.1038/s41598-021-89341-x PMC8113448 33976280

[2] Hafner M, Stepanek M, Taylor J, Troxel WM, van Stolk C (2017). Why sleep matters–the economic costs of insufficient sleep: A cross–country comparative analysis. Rand Health Q.

[3] Burton WN, Pransky G, Conti DJ, Chen CY, Edington DW (2004). The association of medical conditions and presenteeism. J Occup Environ Med.

[4] Suzuki T, Miyaki K, Song Y, Tsutsumi A, Kawakami N, Shimazu A (2015). Relationship between sickness presenteeism (WHO-HPQ) with depression and sickness absence due to mental disease in a cohort of Japanese workers. J Affect Disord.

[5] Dement WC (2005). Sleep extension: Getting as much extra sleep as possible. Clin Sports Med.

[6] Okajima I, Komada Y, Ito W, Inoue Y (2021). Sleep debt and social jetlag associated with sleepiness, mood, and work performance among workers in Japan. Int J Environ Res Public Health.

[7] Wittmann M, Dinich J, Merrow M, Roenneberg T (2006). Social jetlag: Misalignment of biological and social time. Chronobiol Int.

[8] Komada Y, Okajima I, Kitamura S, Inoue Y (2019). A survey on social jetlag in Japan: A nationwide, cross-sectional internet survey. Sleep Biol Rhythms.

[9] Roenneberg T, Pilz LK, Zerbini G, Winnebeck EC (2019). Chronotype and social jetlag: A (Self-) Critical Review. Biology.

[10] Roenneberg T, Allebrandt KV, Merrow M, Vetter C (2012). Social jetlag and obesity. Curr Biol.

[11] Islam Z, Hu H, Akter S, Kuwahara K (2020). Social jetlag is associated with an increased likelihood of having depressive symptoms among the Japanese working population: The Furukawa Nutrition and Health Study. Sleep.

[12] Levandovski R, Dantas G, Fernandes LC, Caumo W (2011). Depression scores associate with chronotype and social jetlag in a rural population. Chronobiol Int.

[13] Ishibashi Y, Shimura A (2020). Association between work productivity and sleep health: A cross-sectional study in Japan. Sleep Health.

[14] Swanson LM, Arnedt JT, Rosekind MR, Belenky G, Balkin TJ, Drake C (2011). Sleep disorders and work performance: Findings from the 2008 national sleep foundation sleep in America poll. J Sleep Res.

[15] Itani O, Kaneita Y, Otsuka Y (2021). A cross-sectional epidemiological study of the relationship between sleep duration, quality, and rhythm and presenteeism in workers. Sleep Biol Rhythms.

[16] Kayaba M, Sasai-Sakuma T, Takaesu Y, Inoue Y (2021). The relationship between insomnia symptoms and work productivity among blue-collar and white-collar Japanese workers engaged in construction/civil engineering work: A cross-sectional study. BMC Public Health.

[17] Baglioni C, Battagliese G, Feige B, Spiegelhalder K, Nissen C, Voderholzer U (2011). Insomnia as a predictor of depression: A meta-analytic evaluation of longitudinal epidemiological studies. J Affect Disord.

[18] McGregor A, Sharma R, Magee C, Caputi P, et al. Explaining variations in the findings of presenteeism research: A meta-analytic investigation into the moderating effects of construct operationalizations and chronic health. J Occup Health Psychol. 2018;23:584–601. 10.1037/ocp0000099 10.1037/ocp0000099 28981302

[19] Suzuki T, Miyaki K, Sasaki Y, Song Y, Tsutsumi A, Kawakami N (2014). Optimal cutoff values of WHO–HPQ presenteeism scores by ROC analysis for preventing mental sickness absence in Japanese prospective cohort. PLoS ONE.

[20] Kitamura S, Hida A, Aritake S, Higuchi S, Enomoto M, Kato M (2014). Validity of the Japanese version of the Munich ChronoType Questionnaire. Chronobiol Int.

[21] Munezawa T, Morin CM, Inoue Y, Nedate K (2009). Development of the Japanese version of the Insomnia Severity Index (ISI–J). Jpn J Psychiatr Treat.

[22] Harvard Medical School 2013. Translated Versions of the HPQ Survey: HPQ Short Form (Japanese)The World Health Organization Health and Work Performance Questionnaire. Retrieved from https://www.hcp.med.harvard.edu/hpq/ftpdir/WMHJ-HPQ-SF_2018.pdf (January 23, 2021.)

[23] Kessler RC, Barber C, Beck A, Berglund P (2003). The World Health Organization Health and Work Performance Questionnaire (HPQ). J Occup Environ Med.

[24] Furukawa TA, Kawakami N, Saitoh M, Ono Y, Nakane Y, Nakamura Y (2008). The performance of the Japanese version of the K6 and K10 in the World Mental Health Survey Japan. Int J Methods Psychiatr Res.

[25] Kessler RC, Barker PR, Colpe LJ, Epstein JF, Gfroerer JC, Hiripi E (2003). Screening for serious mental illness in the general population. Arch Gen Psychiatry.

[26] Chongsuvivatwong V. epiDisplay: Epidemiological data display package. R package version 3.5.0.1. https://CRAN.R-project.org/package=epiDisplay;2018

[27] Lele SR, Keim JL, Solymos P; 2019. ResourceSelection: Resource selection (probability) functions for use-availability Data. R package version 0.3–5. https://CRAN.R-project.org/package=ResourceSelection

[28] Fox J, An WS {R} Companion to Applied Regression. 3rd ed. https://socialsciences.mcmaster.ca/jfox/Books/Companion/;2019. Thousand Oaks CA: Sage

[29] Peduzzi P, Concato J, Kemper E, Holford TR, Feinstein AR (1996). A simulation study of the number of events per variable in logistic regression analysis. J Clin Epidemiol.

[30] Takano Y, Iwano S, Aoki S, Nakano N, Sakano Y (2021). A systematic review of the effect of sleep interventions on presenteeism. BioPsychoSoc Med.

[31] Ministry of Health, Labour and Welfare. National health and nutrition survey; 2020. https://www.mhlw.go.jp/stf/newpage_14156.html Accessed Oct 27, 2020

[32] Burton WN, Chen CY, Schultz AB, Li X (2017). Association between employee sleep with workplace health and economic outcomes. J Occup Environ Med.

[33] Van Dongen HPA, Maislin G, Mullington JM, Dinges DF (2003). The cumulative cost of additional wakefulness: Dose–response effects on neurobehavioral functions and sleep physiology from chronic sleep restriction and total sleep deprivation. Sleep.

[34] Yong M, Fischer D, Germann C, Lang S, Vetter C, Oberlinner C (2016). Are chronotype, social jetlag and sleep duration associated with health measured by Work Ability Index?. Chronobiol Int.

[35] Morin CM, Jarrin DC, Ivers H, Mérette C, LeBlanc M, Savard J (2020). Incidence, persistence, and remission rates of insomnia over 5 years. JAMA Netw Open.

[36] Zhai L, Zhang H, Zhang D (2015). Sleep duration and depression among adults: A meta-analysis of prospective studies. Depress Anxiety.

[37] Fernandez-Mendoza J, Calhoun SL, Bixler EO, Karataraki M, Liao D, Vela-Bueno A (2011). Sleep misperception and chronic insomnia in the general population: Role of objective sleep duration and psychological profiles. Psychosom Med.

